# Adverse Effects of Selected Markers on the Metabolic and Endocrine Profiles of Obese Women With and Without PCOS

**DOI:** 10.3389/fendo.2021.665446

**Published:** 2021-05-26

**Authors:** Mazin H. Daghestani, Maha H. Daghestani, Arjumand Warsy, Afaf El-Ansary, Mohammed A. Omair, Maha A. Omair, Lena M. Hassen, Eman MH. Alhumaidhi, Bashaer Al Qahtani, Abdel Halim Harrath

**Affiliations:** ^1^ Department of Obstetrics & Gynaecology, Medical College, Umm-Al-Qura University, Makkah, Saudi Arabia; ^2^ Zoology Department, Science College, King Saud University, Riyadh, Saudi Arabia; ^3^ Central Laboratory, Center for Science and Medical Studies for Girls, King Saud University, Riyadh, Saudi Arabia; ^4^ Rheumatology Unit, Department of Medicine, College of Medicine, King Saud University, Riyadh, Saudi Arabia; ^5^ Department of Statistics and Operations Research, College of Sciences, King Saud University, Riyadh, Saudi Arabia

**Keywords:** polycystic ovary syndrome, kisspeptin, vitamin D, vascular endothelial growth factor, estradiol, obesity

## Abstract

The aim of the present study, is to investigate the influence of obesity, with and without polycystic ovarian syndrome (PCOS), on the levels of kisspeptin, vitamin D (Vit D), and vascular endothelial growth factor (VEGF) and to explore the relationship between these parameters and endocrine and metabolic variables. The study group included 126 obese Saudi females. Of these 63 were suffering from PCOS while the rest were normo-ovulatory obese women (non-PCOS obese). In the obese PCOS, VEGF was almost four times as high as in the non-PCOS obese, while kisspeptin and Vit D did not differ. A highly significant elevation was recorded in the waist/hip (WHR), cholesterol, LDL-C, fasting glucose, LH, LH/FSH ratio, estradiol (E2), and testosterone, while hip circumference, leptin, progesterone, and sex hormone binding globulin (SHBG) were lower in the obese PCOS subjects. BMI, HDL-C, ghrelin, insulin, and FSH levels did not differ significantly between the two groups. The obese PCOS had the same level of insulin resistance as the non-PCOS group, as judged by QUICK Index. Correlation studies showed a significant negative correlation between kisspeptin and glucose and LH levels, and a positive correlation with LH/FSH ratio in obese PCOS while in the non-PCOS obese, the kisspeptin correlated positively with glucose, and there was no correlation with LH or LH/FSH. VEGF negatively correlated with FSH and positively with LH/FSH ratio in the non-PCOS obese but this was lost in the obese PCOS. PCOS had no effect on the correlation between Vit D and all studied parameters. Multiple regression analysis showed triglyceride as predictor variable for kisspeptin as a dependent variable, while, leptin is a predictor variable for VEGF as a dependent variable. ROC studies showed the highest sensitivity and specificity for VEGF (AOC=1.00), followed by LH/FSH ratio (AOC=0.979). In conclusion, our study shows that PCOS results in significant elevation of VEGF in obese females, while kisspeptin and Vit D levels are not affected. It also leads to elevation in several of the lipid and hormonal abnormalities in the obese females. In addition, PCOS influences relationship between Kisspeptin and VEGF and some parameters such as glucose, LH or FSH and LH/FSH ratio in obese females, but does not affect Vit D relationship with other parameter.

## Introduction

Obesity is associated with several endocrine and metabolic abnormalities, which may result in serious complications such as heart diseases, diabetes mellitus, hypertension, metabolic syndrome and others. It is also a predisposing factor for polycystic ovary syndrome (PCOS) in women of childbearing age, and the prevalence of obesity is as high as 80% in PCOS in some populations (1). In addition, the complications resulting from the synergistic effect of obesity and PCOS may be more deleterious (2). The aim of this study was to investigate the influence of obesity and PCOS, on the levels of kisspeptin, vitamin D (Vit D), and vascular endothelial growth factor (VEGF) and to explore the relationship between these parameters and endocrine and metabolic variables in obese females with and without PCOS.

Polycystic ovarian syndrome is one of the most common endocrine disorders which occurs at a prevalence of 3-10% in different populations (3, 4). It is well documented that some of the common syndromes associated with PCOS are imbalance of sex hormones, insulin resistance, impaired glucose tolerance and dyslipidemias (1, 2, 5, 6). Several parameters influence the symptoms in obesity and PCOS and include kisspeptin, vitamin D (Vit D) and vascular endothelial growth factor (VEGF) (2). Kisspeptins are a group of regulatory neuropeptides that play a crucial role in the control of the hypothalamic–pituitary–gonadal (HPG) axis *via* regulation of gonadotrophin-releasing hormone (GnRH) secretion (7). The *KISS1* gene encodes the kisspeptins and is expressed along with their receptors in the mammalian ovaries, where they help ovulation in sexually mature females (8). Kisspeptins are shown to exert a direct control effect on ovarian functions such as follicular development, oocyte maturation, steroid hormones synthesis, and ovulation and hence, are considered to be essential for reproductive function (9). It is reported that puberty is regulated by the maturation of kisspeptin neurons and by interactions between kisspeptins and leptin. This interaction initiates gonadotropin releasing hormone (GnRH) by the hypothalamus to induce the secretion of luteinising hormone (LH) and follicle stimulating hormone (FSH) (10). Any dysregulation of kisspeptin signalling negatively affect the ovarian function, leading to female reproductive and infertility problems among which is PCOS (11).

The VEGF a homodimeric glycoprotein expressed in granulosa and thecal cells and is known to be involved in the pathophysiology of PCOS (12). It plays an essential role in follicle maturation, quality of oocyte, fertilization and embryo development (2). Impaired levels of VEGF were recorded in the blood or in granulosa lutein cell culture media in *in-vitro* laboratory studies of women with PCOS. It was concluded that VEGF role might be affected with the bioavailability of its soluble receptor, other cytokines and growth factors (13).Vitamin D is a very important regulator of mineral and bone homeostasis and hypovitaminosis D is a worldwide health problem. It has been implemented in the pathophysiology of obesity, insulin resistance and PCOS (14–16). A recent study from Saudi Arabia, reported a high prevalence of Vit D deficiency in Saudi PCOS patients (17). The pathophysiological relevance of insufficient Vit D levels in PCOS has been investigated in several studies, and it is shown that Vit D deficiency is associated with adverse fertility outcomes including PCOS. Recent reports suggest that metabolic, endocrine and fertility aspects in PCOS may benefit from Vit D supplementation (18, 19).

Obesity, especially abdominal and ectopic fat accumulation are major risk factors in the development of a number of chronic diseases among which are diabetes, PCOS, dyslipidemia, hyper-androgenemia, and anovulation (20–22). In a systematic review and meta-analysis, (22) the prevalence of obesity, and central obesity in women with or without PCOS was reported and it was found that the prevalence of overweight, obesity, and central obesity was significantly higher among women with PCOS compared to their matching obese women without PCOS. While a high prevalence of obesity in PCOS patients is clear, the role that kisspeptin, Vit D and VEGF play in the pathophysiology of obese PCOS is not fully understand.

This information initiated our interest to explore the relationship between kisspeptin, VEGF and Vit D, in the etiology of obese PCOS and to investigate their influence on metabolic and endocrine parameters in these patients, since abnormalities in these may lead to cardiovascular and other complications in PCOS.

## Material and Methods

This prospective observational cross-sectional study was conducted at the Department of Obstetrics and Gynecology, Hera Hospital, Makkah, Kingdom of Saudi Arabia. The study protocol was approved by the ethical committee and a total of 126 obese patients attending the out-patient Gynecology Clinic were recruited in the study, after they had signed the informed consent form. Of these, 63 women were suffering from PCOS. The diagnosis of PCOS was made by the clinical co-investigators in the group, and was according to the Rotterdam consensus (23) based on the association of at least two of the three following criteria:

1. Anovulation; presented as low luteal progesterone and normal serum FSH levels (normal range: 1.0–10.0 IU/l).
2. Biochemical signs of raised androgens; elevated serum androgen levels (total testosterone >2 nmol/l), and/or androstenedione >0.15 nmol/l, and/or dehydroepiandrosterone sulphate (DHEAS) >10 mmol/l); LH to FSH ratio >2.
3. Ultrasound criterion of PCO: at least one ovary contained >12 follicles measuring 2–9 mm in diameter and/or increased ovarian volume of at least 10 ml.

The control group consisted of 63 normo-ovulatory obese women. They had regular ovulatory cycles (25–35 days), no endocrine abnormalities, no clinical or biochemical signs of raised androgens, and normal ultrasonic ovarian morphology. The control women were matched with PCOS women for age and body mass index (BMI).

Exclusion criteria for all the subjects included Cushing’s syndrome, pregnancy, hypothyroidism, hyper-prolactinemia, adrenal hyperplasia, current or previous use of anti-androgens, ovulation induction agents, oral contraceptives, glucocorticoids, anti-diabetic and anti-obesity drugs or any hormonal drugs (within the last six months). None of the patients was affected by cardiovascular disorder, neoplastic, metabolic and/or other concurrent medical illness such as hepatic disorders, diabetes, and renal disease. All the subjects were non-smokers and had normal physical activity.

Anthropometric measurements:

For each woman, weight and height were measured to calculate the BMI (weight in kg divided by height in m^2^). Patients with a BMI >29.9 kg/m^2^ were considered obese. Waist circumference (the narrowest circumference between the lower costal margins and the iliac crest) and hip circumference (the maximum circumference at the level of the femoral trochanters) were also measured in the standing position to calculate the waist–hip ratio (WHR).

Blood collection:

During the early follicular phase (2^nd^ or 3^rd^ day), after an overnight fast, 5 ml blood was drawn during the early morning between 07.00 h and 08.00 in plain red-top tubes. For the measurement of 17 OH-progesterone (P), testosterone (T), and sex-hormone binding globulin (SHBG) levels, 5 ml blood samples were extracted on day 20 or 21 of the menstrual cycle. Two ml of blood were drawn in fluoride tubes (grey top) for glucose estimation. All blood samples for each woman were immediately centrifuged, and the serum was stored at -80°C until further analysis.

Measurement of biochemical and hormonal parameters:

The serum was thawed when required for analysis, and used for the estimation of total serum cholesterol, triglycerides, low and high density lipoprotein (HDL-C and LDL-C) by an enzymatic methods using commercial kits (Boehringer Mannheim). Serum was also used for the estimation of basal serum levels of LH, FSH, E2, P, T, SHBG and insulin using specific ELISA Kits (Human, Cat. No.65205.GER).Plasma glucose levels were determined, in the plasma obtained from the blood collected in fluoride tubes, by the glucose oxidase method on a Beckman Glucose Analyzer (Fullerton, CA),. Total ghrelin levels were measured in duplicate using a commercial ghrelin (human) enzyme immunoassay kit (EIA) from (Phoenix Pharmaceuticals, Inc., Belmont, CA, USA), with a lower limit of detection of 0.06 ng/ml and leptin levels were determined using ELISA Kit (Phoenix Pharmaceuticals). Serum VEGF concentration (ng/µl) was evaluated in duplicate using a VEGF enzyme immunoassay (ELISA) kit (R&D Systems, Quantikine Inc., USA) Cat. No. DVE00, and kisspeptin was estimated using ELISA Test Kit from Phoenix Pharmaceuticals Inc., Belmond, CA, following extraction with Phoenix Peptide sep-columns (RK-Sepcol-2). The Vit D level was assessed by ELISA using kits from Eagle Biosciences (Amherst, NH, USA).

### Statistical Analysis

The data collected from the patients and controls was fed on Excel spread sheets and analysed using Statistical Program for Social Sciences (SPSS) (SPSS Inc., Chicago, IL, USA) version 22 for all analyses. Mean, standard deviations (SD), standard error of the mean (SEM), maximum and minimum values, percent changes compared to the control group were obtained. Frequency distribution histograms were plotted. Kisspeptin, Vit D and VEGF were correlated with all studied parameters and Pearson correlation coefficient (r) was obtained. Multiple Regression analysis were conducted. Receiver Operating curves (ROC) were obtained. Comparison was made between the obese PCOS and obese control group using student’s ‘t’ test and p value<0.05 was considered statistically significant.

## Results

**Figure 1:**
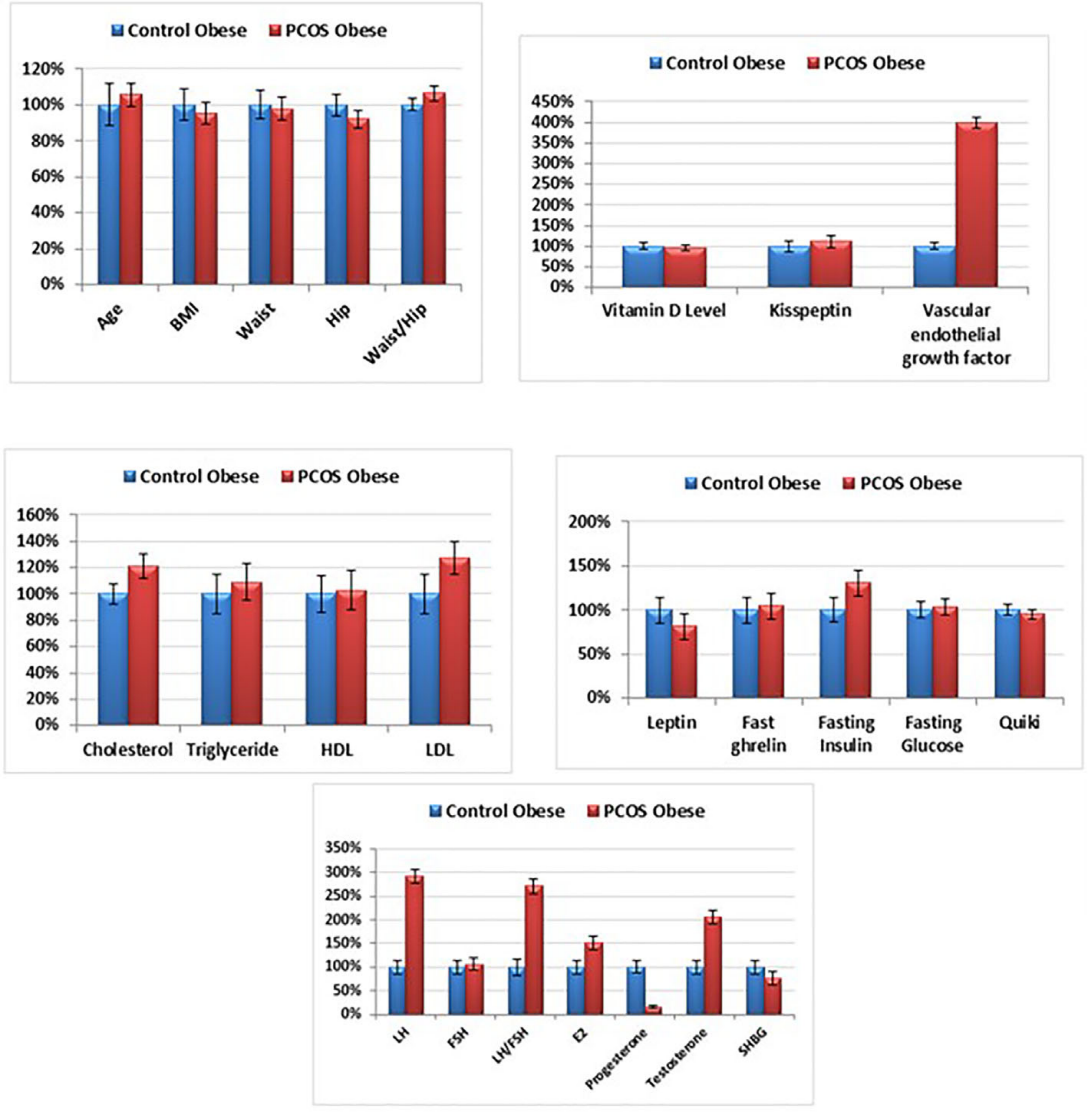
Percentage change of all studied parameters in obese PCOS group compared to the obese control group.

Both the study groups [obese non-PCOS (as controls) and obese PCOS (as patients)] and were BMI and age-matched (**Table 1** and **Figure 1**). High waist/hip ratios were recorded in PCOS patients compared to control group. As presented in **Table 1** and **Figure 1**, the levels of kisspeptin were higher in the PCOS group, but the difference was not significant statistically. Vitamin D levels were the same in both groups, while the levels of VEGF were almost 4 times higher in the PCOS compared to the control group. The PCOS group also suffered from dyslipidaemias, presented as significantly higher cholesterol and LDL-C levels. However, there was non-significant elevation of triglycerides in PCOS, and the HDL level was the same as in the controls. In the endocrines, ghrelin levels were unchanged between the two groups, while, leptin levels were significantly lower in obese PCOS compared to obese control subjects (**Table 1** and **Figure 1**). The PCOS patients had higher levels of fasting insulin, glucose, T, E2, LH and LH/FSH ratio, while hip circumference, progesterone and SHBG were significantly lower and FSH was not changed (**Table 1** and **Figure 1**). To evaluate insulin resistance in the PCOS and control groups, the value of Quantitative Insulin Sensitivity Check Index (QUICKI) (24) was calculated applying the formula:

QUICKI=1/[(log fasting Insulin (mU/ml))+(log fasting glucose (mg/ml))],

**Table 1:** The mean, SD, percentage change minimum and maximum values for the studied parameters in obese PCOS and obese controls, and the significance of the difference between the two groups.

Parameters	Groups	Min.	Max.	Mean ± S.D.	Percent Change	P value*
Age (years)	Control	19.00	36.00	24.40 ± 5.70	100.00%	0.102
	PCOS	19.00	32.00	25.76 ± 3.24	105.60%	
BMI (kg/m2)	Control	30.00	51.50	34.90 ± 6.03	100.00%	0.073
	PCOS	29.00	46.50	33.23 ± 4.14	95.21%	
Waist (cm)	Control	70.00	134.00	96.30 ± 15.30	100.00%	0.408
	PCOS	76.00	134.00	94.27 ± 11.99	97.89%	
Hip (cm)	Control	95.00	150.00	118.56 ± 14.41	100.00%	0.0001
	PCOS	92.00	145.00	109.16 ± 10.82	92.07%	
Waist/Hip	Control	0.63	0.93	0.81 ± 0.06	100.00%	0.0001
	PCOS	0.75	1.15	0.86 ± 0.07	106.59%	
Vitamin D Level (ng/ml)	Control	30.00	67.00	45.65 ± 7.91	100.00%	0.134
	PCOS	30.00	60.00	43.65 ± 6.94	95.62%	
Kisspeptin (fmol/mL)	Control	0.17	0.56	0.38 ± 0.10	100.00%	0.110
	PCOS	0.20	0.72	0.42 ± 0.12	110.75%	
VEGF (nmol/L)	Control	70.00	132.00	95.48 ± 16.11	100.00%	0.0001
	PCOS	176.00	512.00	380.60 ± 91.10	398.64%	
Cholesterol (mmol/L)	Control	2.90	5.20	3.89 ± 0.58	100.00%	0.0001
	PCOS	2.70	6.40	4.72 ± 0.90	121.17%	
Triglyceride (mmol/L)	Control	0.53	1.50	1.03 ± 0.30	100.00%	0.090
	PCOS	0.50	1.80	1.13 ± 0.32	109.18%	
HDL (mmol/L)	Control	0.59	2.10	1.11 ± 0.30	100.00%	0.581
	PCOS	0.60	2.00	1.14 ± 0.34	102.85%	
LDL-C (mmol/L)	Control	0.70	3.60	2.13 ± 0.62	100.00%	0.0001
	PCOS	1.10	4.00	2.72 ± 0.68	127.39%	
Leptin (ngl/ml)	Control	25.00	85.00	39.73 ± 11.44	100.00%	0.0001
	PCOS	12.00	56.00	32.29 ± 9.46	81.26%	
Fast ghrelin (ngl/ml)	Control	0.16	0.54	0.33 ± 0.10	100.00%	0.377
	PCOS	0.14	0.60	0.34 ± 0.10	104.78%	
Fasting Insulin (pmol/L)	Control	47.00	140.00	87.56 ± 23.78	100.00%	0.185
	PCOS	53.30	207.50	114.36 ± 32.04	130.61%	
Fasting Glucose (mmol/l)	Control	3.80	5.80	4.92 ± 0.50	100.00%	0.033
	PCOS	4.10	6.60	5.11 ± 0.45	103.71%	
QUICKI	Control	0.297	0.411	0.33 ± 0.03	100.00%	0.258
	PCOS	0.283	0.417	0.32 ± 0.03	95.14%	
LH (IU/L)	Control	1.80	7.90	4.62 ± 1.31	100.00%	0.0001
	PCOS	6.30	23.00	13.46 ± 3.95	291.21%	
FSH (IU/L)	Control	2.30	7.30	4.84 ± 1.39	100.00%	0.231
	PCOS	2.20	9.60	5.15 ± 1.44	106.26%	
LH/FSH	Control	0.54	1.59	1.01 ± 0.33	100.00%	0.0001
	PCOS	1.32	4.29	2.74 ± 0.84	270.92%	
E2 (pmol/L)	Control	95.00	276.60	143.20 ± 40.68	100.00%	0.0001
	PCOS	125.70	394.70	217.16 ± 61.95	151.65%	
Progesterone (nmol/L)	Control	6.20	30.00	16.15 ± 4.32	100.00%	0.0001
	PCOS	1.30	4.20	2.50 ± 0.78	15.46%	
Testosterone (nmol/L)	Control	0.54	2.00	1.37 ± 0.40	100.00%	0.0001
	PCOS	0.91	4.85	2.82 ± 0.81	206.27%	
SHBG (nmol/L)	Control	23.00	68.00	37.67 ± 10.40	100.00%	0.001
	PCOS	18.00	45.00	28.65 ± 8.47	76.06%	

*P value between Control Obese group and PCOS Obese group using Independent Students ‘t’ test.

The value of QUICKI for the PCOS and controls is presented in **Table 1**.

The normal frequency distribution histogram of those parameters, were significantly different between the two groups (**Figure A, Supplementary Material**). The normal distribution of kisspeptin, demonstrates the higher levels of this variable in obese PCOS. VEGF levels were significantly higher in all PCOS and all patients recorded levels higher than 200 nmole/L, with levels as high as 500-600 nmole/L in some patients, while the highest level recorded in the control group was only 140 nmole/L. Vitamin D levels did not differ between the two groups. It was observed that 46/63 of the PCOS group recorded fasting insulin level greater than 100 pm/L compared to only 13/63 in control group. Regarding the LH, and LH/FSH, 53/63 patients recorded LH level higher than 10 IU/L, against none in the control group. This was reflected as remarkably higher ratio of LH/FSH. Estradiol frequency distribution shows that 38/63 of PCOS, and 5/63 obese controls recorded E2 levels greater than 200 pmole/L. Progesterone levels were significantly lower in the PCOS group. The figure also shows that while PCOS patients (63/63) recorded progesterone level lower than 5 nmole/L, all controls recorded much higher levels. While, testosterone levels were significantly higher in the obese PCOS compared to the obese controls, where 54/63 PCOS patients had testosterone levels higher than 2.0 nmole/L *vs*. only 3/63 in control group.

**Figure 2:**
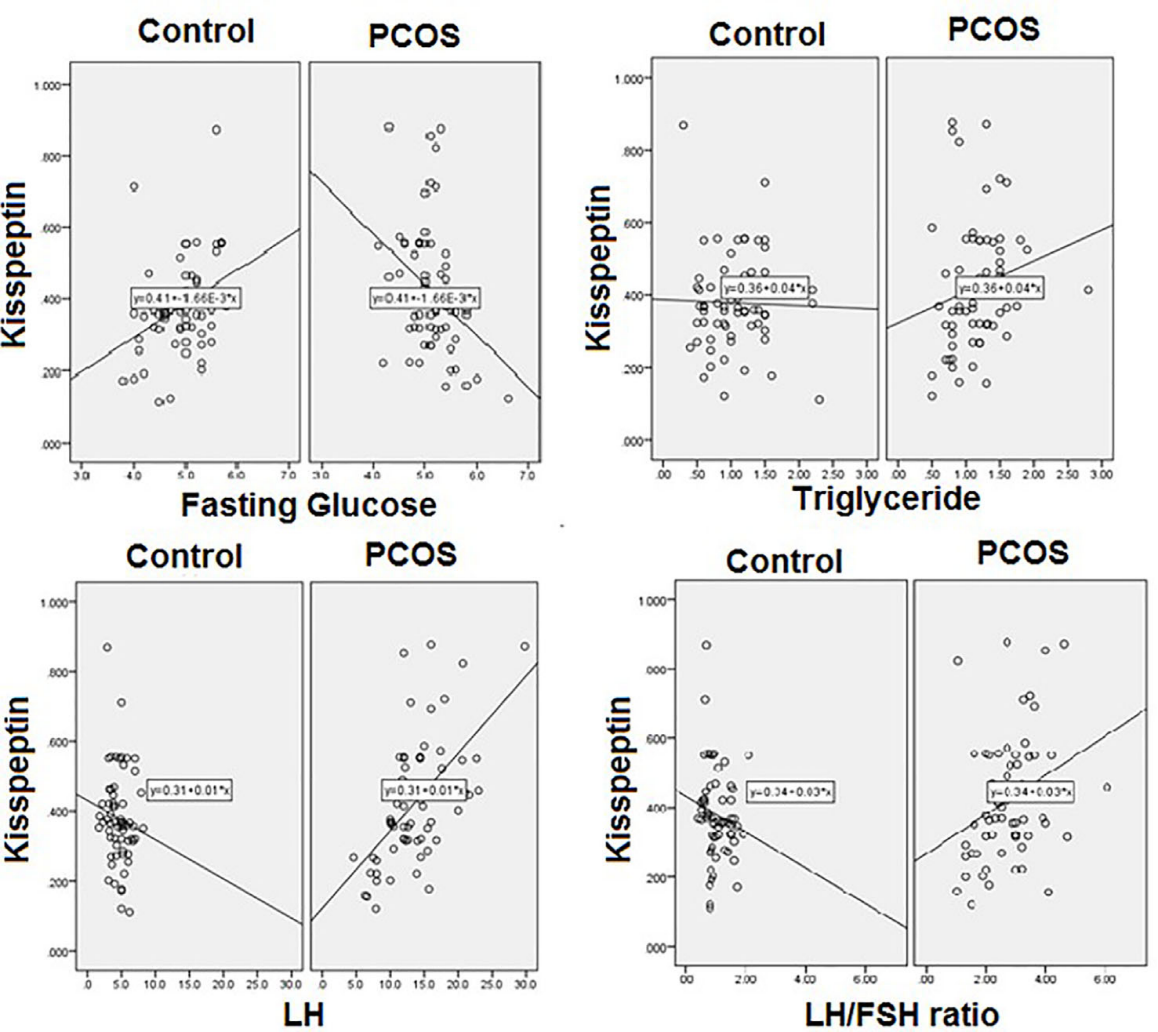
Correlation between kisspeptin and glucose, triglycerides, LH, LH/FSH ratio.

Pearson’s correlation studies between vitamin D, kisspeptin and VEGF, show several interesting facts (**Tables 2** **–** **4**). In the PCOS group kisspeptin correlated negatively with glucose, while in the control group the correlation was positive and significant (**Table 2** and **Figure 2**). It also correlated positively with LH, LH/FSH ratio and triglycerides in the PCOS group, while negatively with these parameters in the controls. Interestingly, it correlated positively with BMI, waist and hip circumference in the PCOS, though the correlation was not statistically significant, while, there was almost no correlation in the controls (**Table 2** and **Figure 2**). Vitamin D did not correlate significantly with any parameter, but showed a positive non-significant correlation with HDL-C in the control group and negative correlation in the PCOS (**Table 3**). Finally, VEGF demonstrated significant negative correlations with FSH and positive correlation with LH/FSH ratio in the control group, but the correlation was lost in the PCOS (**Table 4**). With estrogen levels, it correlated positively in controls and negatively in PCOS, with leptin it showed a positive correlation in controls and negative in PCOS and with ghrelin it did not correlate in controls, but showed a positive correlation in the PCOS, though the correlation was not significant (**Table 4**).

**Table 2:** Correlation between kisspeptin and the studied parameters in the obese PCOS patients and obese control group.

Correlation between Kisspeptin and	Obese Control		Obese PCOS	
	r	p	r	p
Age (years)	0.015	0.907	0.039	0.760
BMI (kgm2)	-0.031	0.808	0.228	0.072
Waist	-0.111	0.387	0.192	0.132
Hip	-0.086	0.500	0.233	0.066
WH ratio	-0.087	0.499	0.003	0.983
Cholesterol (mmolL)	0.031	0.809	0.102	0.426
Triglyceride (mmolL)	-0.028	0.829	0.195	0.125
HDL (mmolL)	-0.106	0.408	-0.184	0.148
LDL (mmolL)	0.055	0.670	0.102	0.425
Leptin nglml	-0.075	0.561	-0.109	0.394
Fast ghrelin nglml	-0.094	0.464	-0.07	0.584
Fasting Insulin (pmolL)	0.071	0.582	0.064	0.617
Fasting Glucose	0.358^**^	0.004	-0.358^**^	0.004
LH (IUL)	-0.122	0.339	0.571^**^	0.000
FSH (IUL)	0.073	0.570	-0.010	0.935
LHFSH ratio	-0.152	0.233	0.306^*^	0.015
E2 (pmolL)	-0.229	0.070	0.100	0.433
Progesterone (nmolL)	0.019	0.882	-0.128	0.318
Testosterone (nmolL)	-0.185	0.146	-0.003	0.978
SHBG (nmolL)	0.224	0.077	-0.246	0.052
Vitamin D Level ng/ml	0.053	0.678	0.129	0.314
VEGF nmol/L	-0.082	0.523	-0.161	0.206

*Correlation is significant at the 0.05 level (2-tailed).

**Correlation is significant at the 0.01 level (2-tailed).

**Table 3:** Correlations between Vit D and the studied parameters in obese PCOS and obese non-PCOS controls.

Correlation between Vitamin D Level and	Obese Controls		Obese PCOS	
	r	p	r	p
Age (years)	-0.102	0.428	0.188	0.140
BMI (kgm2)	-0.083	0.520	-0.119	0.353
Waist	-0.210	0.098	-0.085	0.510
Hip	-0.145	0.258	-0.177	0.165
WH ratio	-0.215	0.091	0.097	0.450
Cholesterol (mmolL)	0.040	0.757	0.028	0.826
Triglyceride (mmolL)	0.120	0.347	0.065	0.611
HDL (mmolL)	0.178	0.162	-0.021	0.867
LDL (mmolL)	-0.147	0.250	0.033	0.798
Leptin nglml	-0.146	0.255	0.102	0.425
Fast ghrelin nglml	-0.033	0.800	-0.031	0.809
Fasting Insulin (pmolL)	0.052	0.687	0.121	0.344
Fasting Glucose	0.042	0.744	-0.121	0.344
QUICKI				
LH (IUL)	0.120	0.349	0.163	0.201
FSH (IUL)	0.118	0.357	0.086	0.505
LHFSH ratio	-0.114	0.374	0.067	0.604
E2 (pmolL)	0.078	0.543	-0.173	0.175
Progesterone (nmolL)	-0.021	0.869	-0.171	0.180
Testosterone (nmolL)	0.118	0.358	-0.101	0.429
SHBG (nmolL)	-0.002	0.985	-0.008	0.950
Kisspeptin fmol/mL	0.053	0.678	0.129	0.314
VEGF nmol/L	-0.077	0.547	-0.112	0.381

**Table 4:** Correlations between VEGF and the studied parameters in obese PCOS and obese non-PCOS controls.

Correlation between VEGF and	Control		PCOS Patients	
	r	p	r	p
Age (years)	-0.117	0.362	0.144	0.259
BMI (kgm2)	0.106	0.408	-0.178	0.164
Waist	0.141	0.270	-0.102	0.428
Hip	0.221	0.081	-0.117	0.362
WH ratio	-0.036	0.780	-0.036	0.778
Cholesterol (mmolL)	-0.141	0.270	-0.107	0.404
Triglyceride (mmolL)	-0.231	0.069	-0.076	0.555
HDL (mmolL)	0.014	0.912	-0.120	0.351
LDL (mmolL)	-0.103	0.423	-0.120	0.348
Leptin nglml	0.138	0.280	-0.194	0.127
Fast ghrelin nglml	-0.091	0.477	0.145	0.256
Fasting Insulin (pmolL)	-0.097	0.451	-0.062	0.628
Fasting Glucose	-0.039	0.760	0.022	0.863
LH (IUL)	0.174	0.172	-0.182	0.154
FSH (IUL)	-.0295^*^	0.019	-0.208	0.101
LHFSH ratio	0.458^**^	0.000	0.106	0.406
E2 (pmolL)	0.232	0.067	-0.212	0.095
Progesterone (nmolL)	0.183	0.151	0.035	0.787
Testosterone (nmolL)	0.187	0.143	0.240	0.058
SHBG (nmolL)	-0.163	0.203	-0.059	0.645
Vitamin D Level ng/ml	-0.077	0.547	-0.112	0.381
Kisspeptin fmol/mL	-0.082	0.523	-0.161	0.206

*Correlation is significant at the 0.05 level (2-tailed).

**Correlation is significant at the 0.01 level (2-tailed).

With multiple linear regression analysis using vitamin D as dependent variable, there were no significant correlations with any of the measured parameters as independent variables, except kisspeptin revealed a positive correlation with triglycerides (**Table 5**) in PCOS group but not in control participants. Furthermore, VEGF recorded positive correlation with leptin in the PCOS (**Table 6**).

**Table 5:** Multiple Regression using Stepwise method for Kisspeptin as a dependent variable for PCOS Obese.

Predictor Variable	Coefficient	P value	Adjusted R square	Model	
				F value	P value
Triglyceride	0.105	0.031	0.059	4.869	0.031

**Table 6:** Multiple Regression using Stepwise method for VEGF as a dependent variable for Control Obese.

Predictor Variable	Coefficient	P value	Adjusted R square	Model	
				F value	P value
Leptin (ngl/ml)	0.420	0.018	0.074	5.959	0.018

**Table 7** shows the ROC analysis of the measured parameters. Among the studied variables, LH, LH/FSH, estrogen, testosterone, progesterone, and VEGF recorded the highest AUCs range between (0.8-1), followed by Waist/hip, cholesterol, LDL, SHBG, and fasting blood insulin with AUCs range of (0.719-0.76). The highest AUC was observed for VEGF.

**Table 7:** The AOC value, sensitivity and specificity of all parameters in obese PCOS group.

	Area under the curve	Cut-off value	Sensitivity %	Specificity %	P value
Vitamin D Level (ng/ml)	0.572	48.500	77.8%	34.9%	0.164
Kisspeptin (fmol/mL)	0.590	0.428	49.2%	71.4%	0.082
VEGF (nmol/L)	1.000	154.000	100.0%	100.0%	0.001
Age (years)	0.642	22.500	82.5%	57.1%	0.006
BMI (kg/m2)	0.563	37.250	84.1%	30.2%	0.221
Waist	0.524	99.500	77.8%	38.1%	0.636
Hip	0.695	121.500	92.1%	39.7%	0.001
Waist/Hip	0.719	0.833	68.3%	68.3%	0.001
Cholesterol (mmol/L)	0.775	4.050	77.8%	66.7%	0.001
Triglyceride (mmol/L)	0.587	1.079	63.5%	60.3%	0.091
HDL (mmol/L)	0.504	0.930	36.5%	81.0%	0.934
LDL (mmol/L)	0.742	2.250	74.6%	65.1%	0.001
Leptin (ngl/ml)	0.672	40.500	82.5%	42.9%	0.001
Fast ghrelin (ngl/ml)	0.547	0.280	79.4%	30.2%	0.364
Fasting Insulin (pmol/L)	0.760	106.900	73.0%	81.0%	0.001
Fasting Glucose	0.590	4.650	85.7%	30.2%	0.080
LH (IU/L)	0.996	7.050	96.8%	98.4%	0.001
FSH (IU/L)	0.550	3.950	87.3%	30.2%	0.332
LH/FSH	0.979	1.604	90.5%	100.0%	0.001
E2 (pmol/L)	0.850	200.250	61.9%	92.1%	0.001
Progesterone (nmol/L)	1.000	5.200	100.0%	100.0%	0.001
Testosterone (nmol/L)	0.939	2.040	85.7%	100.0%	0.001
SHBG (nmol/L)	0.748	24.500	42.9%	93.7%	0.001

## Discussion

In this study we assessed the association of vitamin D, kisspeptin and VEGF with metabolic and endocrine factors in obese women with and without PCOS. Present study showed that W/H ratio, total cholesterol, LDL-C, fasting glucose, and fasting insulin are higher in PCOS obese women compared to the non- PCOS obese controls. In addition, LH, LH/FSH, E2, and testosterone, as endocrine-related markers show highly significant elevation in PCOS, while progesterone and SHBG show highly significant decrease in PCOS patients compared to control participants.

**Table 1** demonstrates the mean ± S.D of all the measured parameters, and shows that the two groups matched in age, BMI, and waist circumference, ascertaining the matching of the groups enrolled for the study. However, the significantly lower hip, and higher waist/hip ratio in PCOS compared to obese control, confirms the contribution of abdominal fat as an etiological mechanism in PCOS. Moreover, the reported increase of fasting glucose and insulin in PCOS obese patients compared to obese controls (**Table 1** and **Figure 1**) can help to suggest that abdominal fat has a critical role in developing hyperinsulinemia in PCOS patients. Insulin resistance, as judged from the value of QUICKI (24) showed that the insulin resistance was the same in both PCOS obese and non-PCOS obese female, clearly showing that it is obesity that affects insulin resistance and PCOS does not. Since studies have shown higher insulin resistance in obesity compared to normal weight, and since both our study groups were obese, we suggest that in our study group the PCOS was not playing a role in elevating insulin resistance. This can find support in several studies which recorded that most PCOS patients exhibit an abdominal form of obesity, and that increased visceral fat may be the cause or the early consequence of their insulin resistance in the obese PCOS patients (15–28). The importance of W/H ratio as prognostic marker further finds support in a recent study of (29) in which they reported that on-treatment, loss of abdominal excess fat, measured as lower W/H ratio was followed by more post-treatment ovulations in adolescent girls with PCOS.

Among the cardiovascular related markers, while triglycerides showed a non-significant elevation, cholesterol and LDL-C were significantly higher in the obese PCOS patients compared to obese control group. Since hypercholesterolemia and high LDL are markers for cardiovascular disease in obesity, we suggest that presence of PCOS in obese females may further increase the risk. Our result is not consistent with the recent study of (30) in which they reported both hypertriglyceridemia and hypercholesterolemia as markers of dyslipidemias that might lead to cardiovascular disease in PCOS patients. We suggest that PCOS can be described as a metabolic disease, which carry important health risks such as hyperinsulinemia, and dyslipidemias. Therefore, strategies to prevent, early diagnose, timely treatment are recommended in order to limit the damage in the course of PCOS progression.

The highly significant increase of LH, LH/FSH, E, and testosterone concomitant with the significant decrease of progesterone in PCOS women compared to control, demonstrate the presence of functional ovarian hyper-androgenism (FOH) as etiological mechanism in PCOS. This is in line with the results of a recent study in which it was reported (31) reported that two-thirds of PCOS demonstrate FOH detectable by testosterone elevation after suppression of adrenal androgen production.

The significant increase of testosterone reported in the present study can be explained in relation to the significant decrease of sex hormone-binding globulin (SHBG) as an important regulatory factor in androgen action and metabolism. In the present study, the significantly lower levels of SHBG in PCOS obese patients compared to control participants, can be easily related to hyperinsulinemia, and increase in testosterone level. These results are supported by several studies which report that in obese patients, levels of SHBG are suppressed by testosterone, hyperinsulinemia, and hyperglycemia. As shown all three of these endocrine and metabolic characteristics were present in the PCOS patients enrolled in the present study (32–34).

In this study, kisspeptin was higher in the obese PCOS group compared to the obese controls, but not significantly. These results contradict some previous reports which show significantly elevated kisspeptin in PCOS compared to controls (35, 36), but are in line with others which report no difference between the two groups (35, 37). These conflicting reports could be due to differences in the BMI in the different studies, however, some studies (19, 26) reported that kisspeptin were higher in PCOS regardless of BMI. Our correlation studies between kisspeptin and the studied parameters revealed some interesting differences between the PCOS and control group. The positive association between kisspeptin and triglycerides, LH and LH/FSH ratio, was not seen in our obese control group. Positive correlation between kisspeptin and LH has been reported in other studies (35, 37). Hypersecretion of LH is a frequent endocrinological finding of PCOS, and it is suggested that this derangement might be in close relationship with hypothalamic kisspeptin expression (38). This has led to the suggestion that kisspeptin antagonist therapy may be useful for the treatment of PCOS (39). Furthermore, in the present study, kisspeptin showed a positive association with waist and hip circumference and BMI, though the correlation was not significant statistically, but it clearly demonstrated that in PCOS, higher levels of kisspeptin are more damaging. Hence, these results further second the use of kisspeptin antagonist therapy, for obese PCOS. It is well accepted that the reproductive and metabolic functions are highly integrated and that kisspeptin has a regulatory role in food intake, glucose homeostasis, and insulin secretion (40–42). Other studies have reported negative correlation between kisspeptin and FSH, testosterone (36), but in the present study, such association was not documented.

A higher prevalence of Vit D deficiency in PCOS compared to controls has been reported frequently (43). Interestingly, our present study showed that the Vit D levels were not different in the two studied groups and there was no Vit D deficiency, defined as levels <30 ng/ml. The role of Vit D has been investigated in several studies. In the animal models, the role played by Vit D in reproductive physiology is quite well documented, but in humans there are inconsistent reports. However, the regulatory role of Vit D, in PCOS-related aspects, such as ovulatory dysfunction, insulin resistance, hyperandrogenism, and metabolic syndrome are well defined and it is shown in some studies that treatment with Vit D supplementation may be beneficial (44), others have failed to show any benefit (45).

Increased stromal vascularity is observed in PCOS and it is suggested that there is a dysregulation of multiple angiogenic factors including VEGF. Angiogenesis is reported to be a major player in leading to the dynamic changes that take place during the normal ovarian cycle (46). The angiogenic factor dysregulation may play a role in the pathophysiology of PCOS and may be among the different factors contributing to the problems commonly seen in women with PCOS, such as subfertility, ovulatory dysfunction, and ovarian hyperstimulation syndrome (47). In the present study the VEGF levels in obese PCOS were four times as high as in the non-PCOS obese women. These results were in line with several studies reporting elevated levels of VEGF in PCOS (13, 48). The mechanisms involved in leading to elevated VEGF levels have been discussed and include genetic variations in the VEGFA gene (49), increased number of actively secreting granulosa lutein cells, and increased secretory capacity of each granulosa cell (49). Interestingly, dietary supplementation of Vit D has been shown to significantly decrease VEGF level (45). Studies at the gene level are required to identify the possible genetic causes of PCOS in Saudi population.

An important point in this study, that we want to comment on, is that anti-Mullerian hormone (AMH), was not included in the battery of tests that we performed during this study. AMH, is a peptide growth factor of the transforming growth factor-β family, and is produced by granulosa cells in ovarian follicles, and is used as a test to assess a women’s egg count and ovarian reserves (50). It is an expensive test and our grant budget could not have afforded it. Furthermore, it is used at individual levels when a woman wants to find out chances of her getting pregnant. Since we were not exploring this factor, we had not considered including this test.

In conclusion, this study has shown that obese PCOS have several metabolic and hormonal abnormalities when compared with obese non-PCOS controls. Kisspeptin is higher in PCOS, but not significantly, and correlates positively with LH, LH/FSH ratio, and triglycerides. It also has a positive though non-significant association with BMI, waist and hip circumference. These finding lead us to propose possible therapeutic interventions to treat PCOS, that will lead to decrease in kisspeptin level. Vitamin D do not show differences between obese PCOS and obese control, while VEGF is the most predictive factors and is significantly elevated in obese PCOS.

## Data Availability Statement

The raw data supporting the conclusions of this article will be made available by the authors, without undue reservation.

## Ethics Statement

This prospective observational cross-sectional study was approved by the ethical committee at the Department of Obstetrics and Gynecology, Hera Hospital, Makkah, Kingdom of Saudi Arabia. The patients/participants provided their written informed consent to participate in this study.

## Author Contributions

MazD, MahD, AW, AfaE, MO, and AH designed the study and drafted the manuscript. MazD, MahD, and MO performed the practicle section. MazD and MO selected the patients and prepared the questionnaire. MahO and AW performed the statistical analysis. All authors contributed to the article and approved the submitted version.

## Conflict of Interest

The authors declare that the research was conducted in the absence of any commercial or financial relationships that could be construed as a potential conflict of interest.
