# Supplementary material for: Adverse Effects of Selected Markers on the Metabolic and Endocrine Profiles of Obese Women With and Without PCOS
Source: Front Endocrinol (Lausanne). 2021 May 26;12:665446. doi: 10.3389/fendo.2021.665446 (PMC8188979; doi:10.3389/fendo.2021.665446)
Supplement: Supplementary file 1 [file DataSheet_1.docx]

**Supplementary Figures**


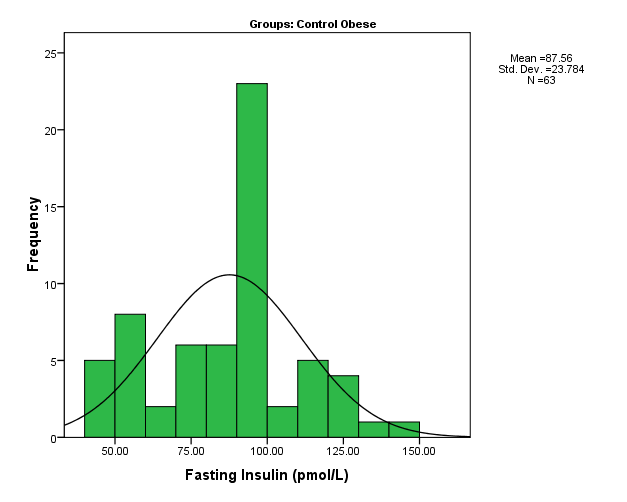

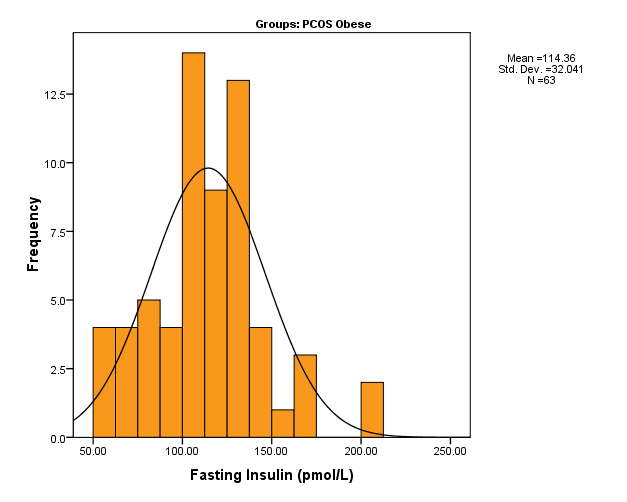


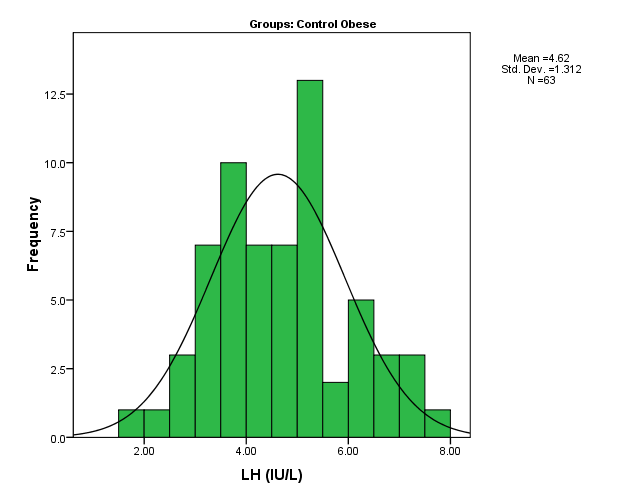

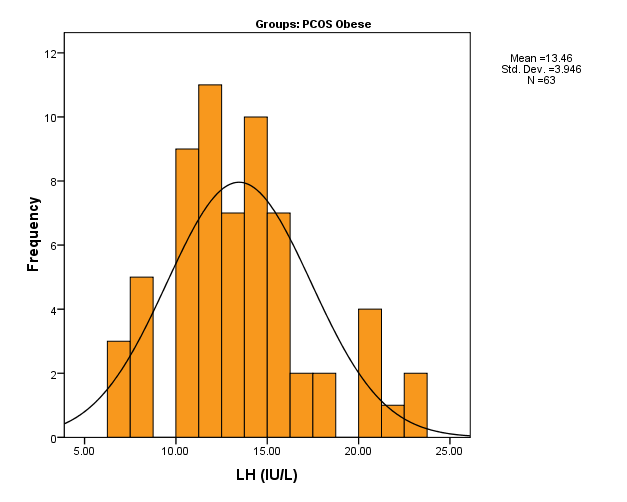


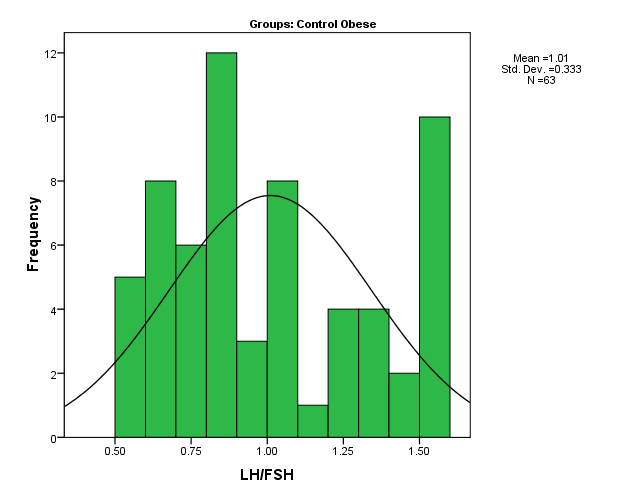

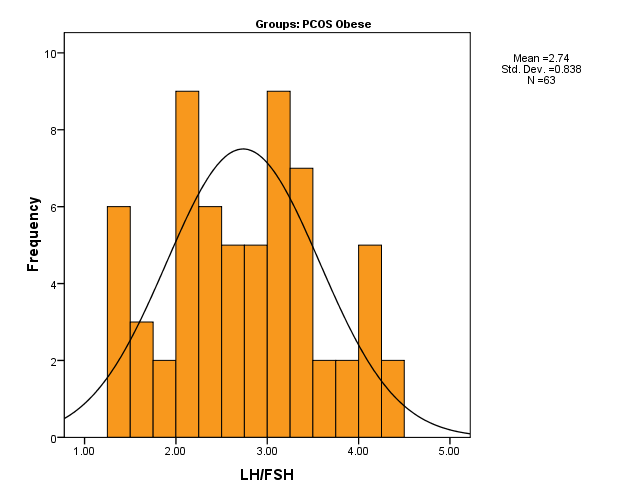


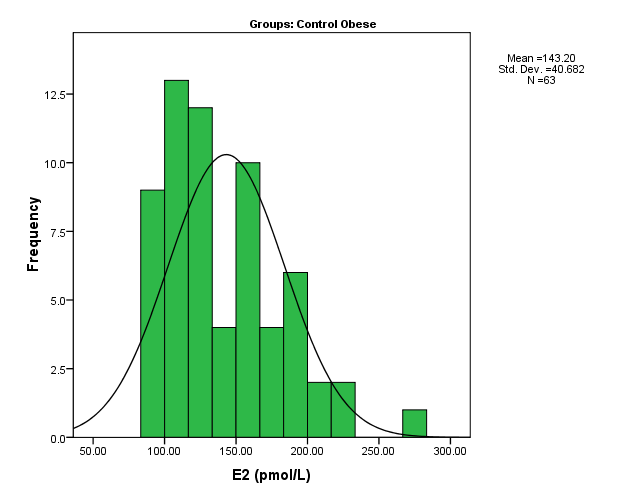

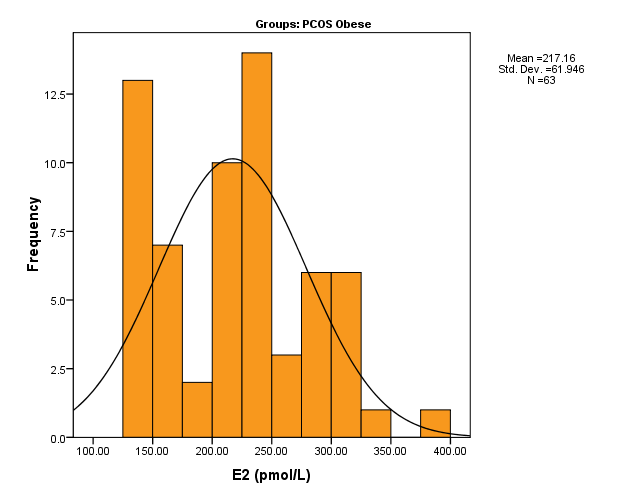


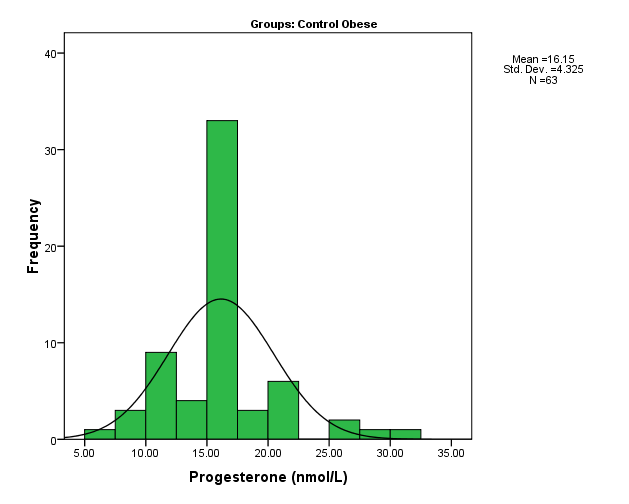

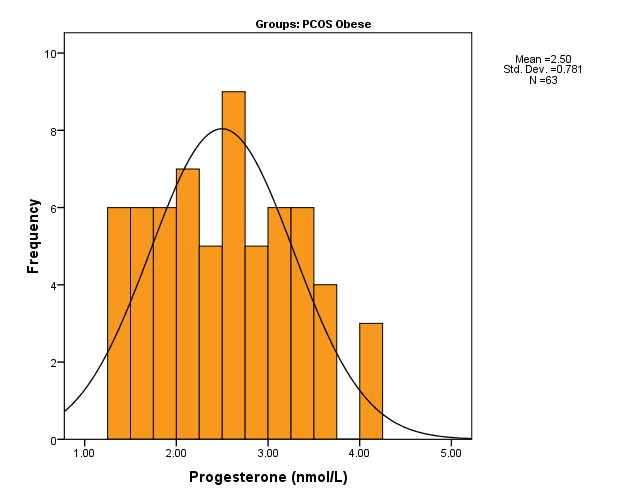


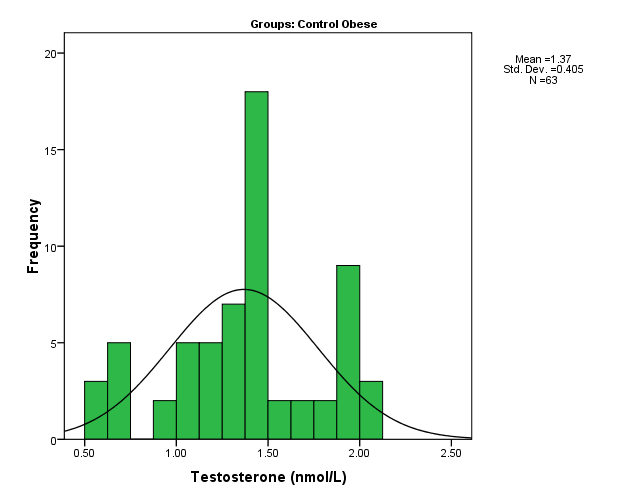

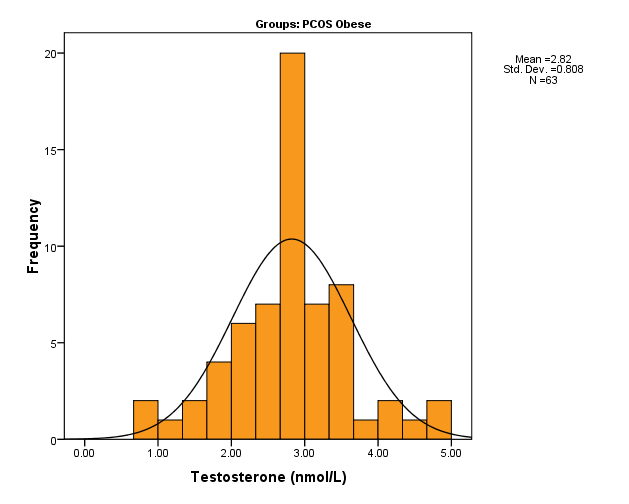


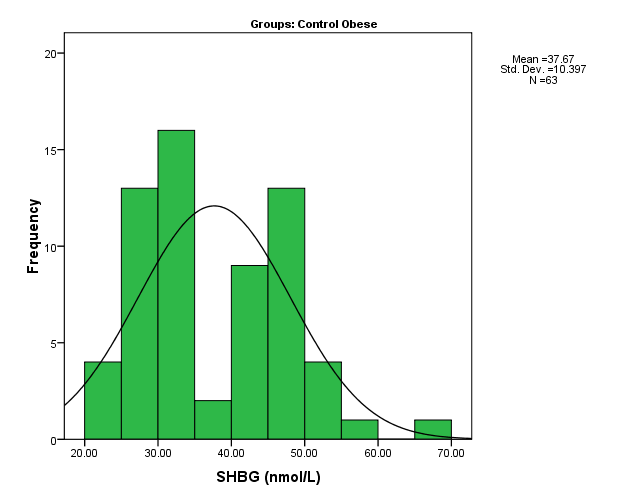

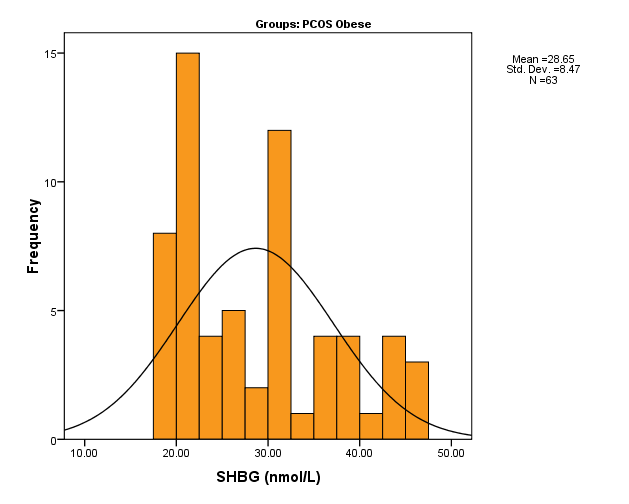


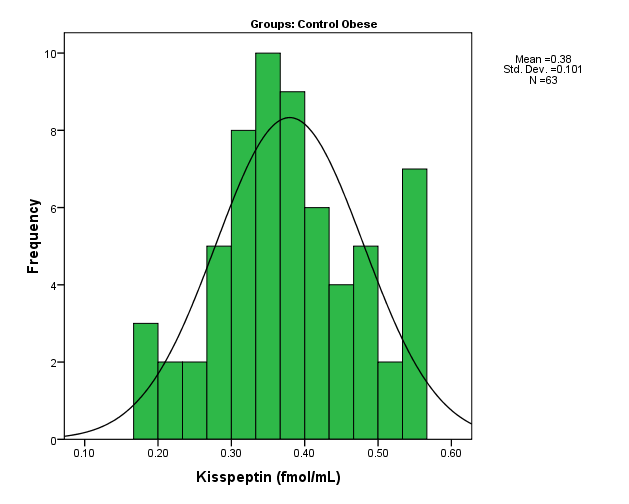

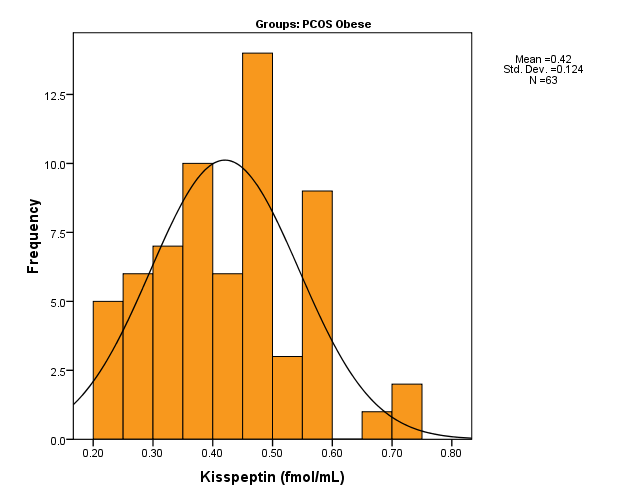


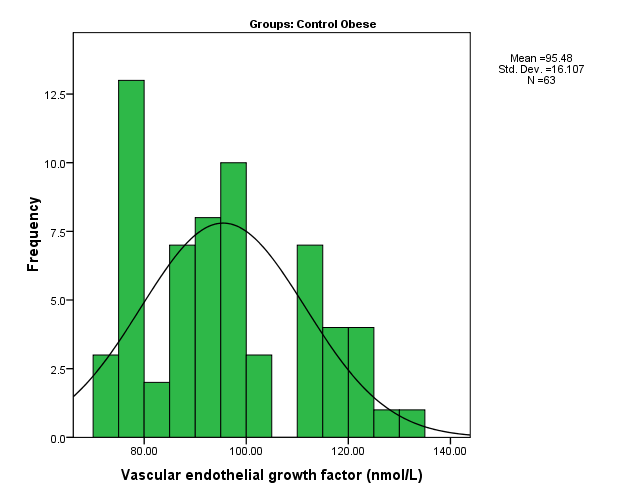

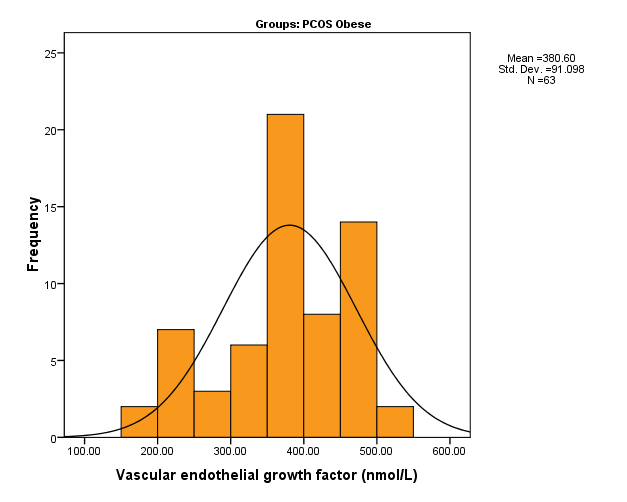


**Fig. 2: Normal frequency distribution histograms of the significantly different parameters in the obese control group and obese PCOS group**


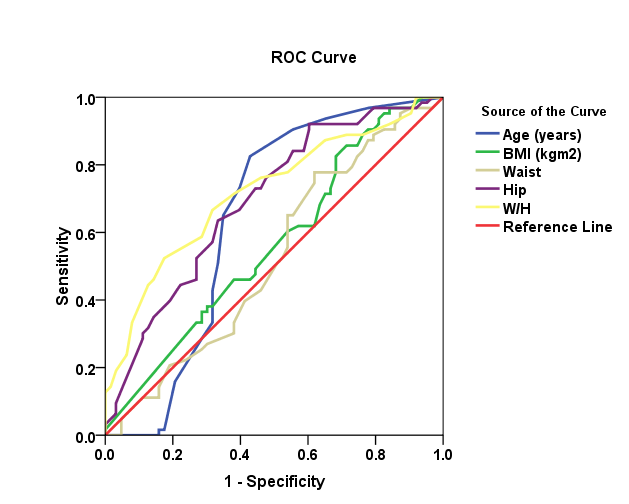


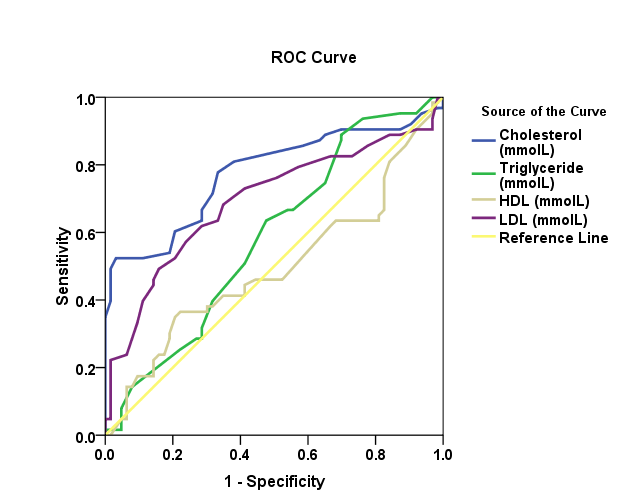


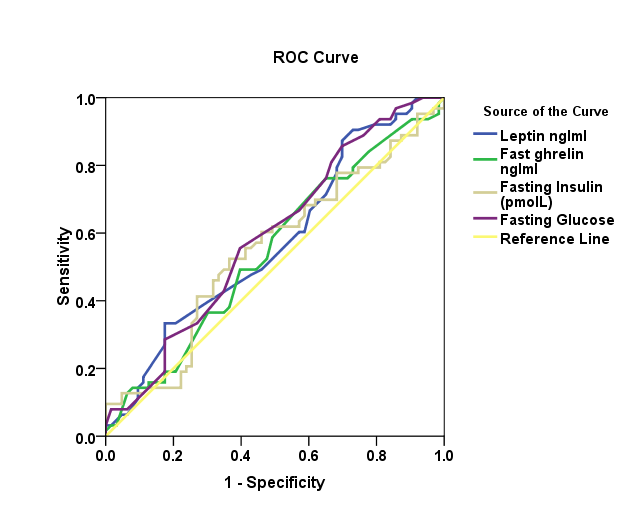


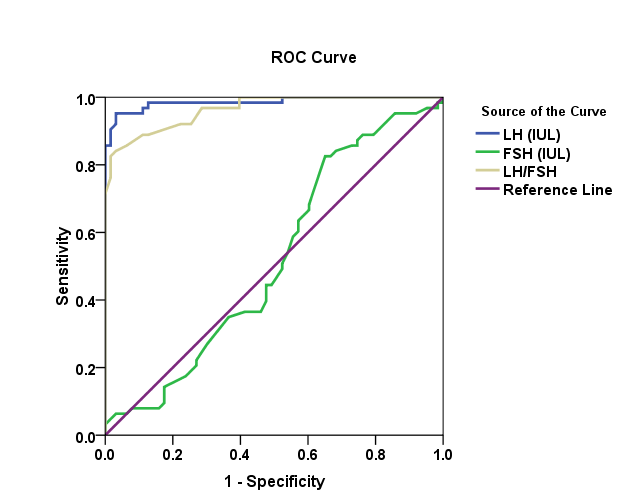


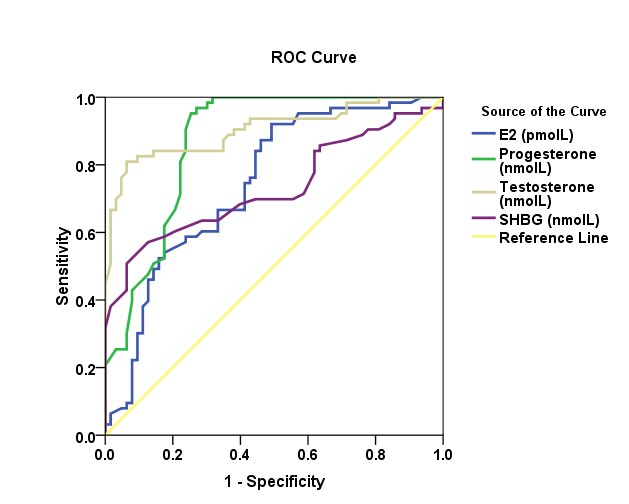


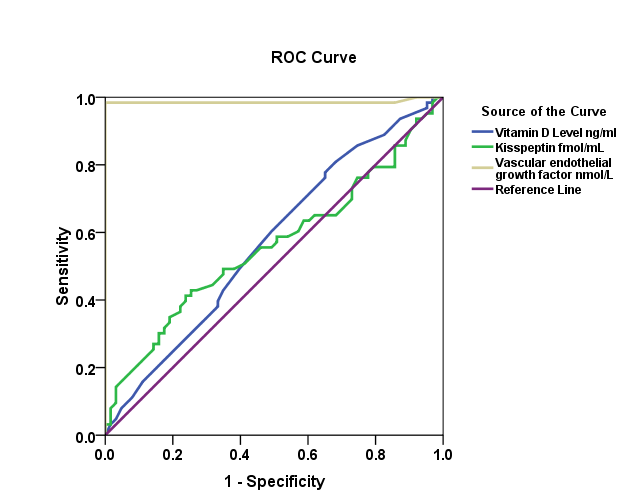


**Figure 2A: Receiver Operating Curves (ROC) for the studied parameters.**
